# Improved analysis of CRISPR fitness screens and reduced off-target effects with the BAGEL2 gene essentiality classifier

**DOI:** 10.1186/s13073-020-00809-3

**Published:** 2021-01-06

**Authors:** Eiru Kim, Traver Hart

**Affiliations:** 1 Department of Bioinformatics and Computational Biology, The University of Texas MD Anderson Cancer Center, Houston, TX USA; 2 Department of Cancer Biology, The University of Texas MD Anderson Cancer Center, Houston, TX USA

## Abstract

**Background:**

Identifying essential genes in genome-wide loss-of-function screens is a critical step in functional genomics and cancer target finding. We previously described the Bayesian Analysis of Gene Essentiality (BAGEL) algorithm for accurate classification of gene essentiality from short hairpin RNA and CRISPR/Cas9 genome-wide genetic screens.

**Results:**

We introduce an updated version, BAGEL2, which employs an improved model that offers a greater dynamic range of Bayes Factors, enabling detection of tumor suppressor genes; a multi-target correction that reduces false positives from off-target CRISPR guide RNA; and the implementation of a cross-validation strategy that improves performance ~ 10× over the prior bootstrap resampling approach. We also describe a metric for screen quality at the replicate level and demonstrate how different algorithms handle lower quality data in substantially different ways.

**Conclusions:**

BAGEL2 substantially improves the sensitivity, specificity, and performance over BAGEL and establishes the new state of the art in the analysis of CRISPR knockout fitness screens. BAGEL2 is written in Python 3 and source code, along with all supporting files, are available on github (https://github.com/hart-lab/bagel).

## Background

The landscape of preclinical studies to identify novel cancer targets has been fundamentally altered by the development of high-throughput genome-wide CRISPR knockout screens [1–3]. The CRISPR-Cas9 system offers significant advantages in specificity and effectiveness of gene knockout [3, 4] over the shRNA knock-down technology that preceded it. Genome-scale knockout screens enable the unbiased identification of genes whose disruption impedes proliferation compared to wildtype cells (“essential genes”), and curation of pan- and context-dependent essential genes is being exploited to identify potential drug targets for specific tumor genotypes [3, 5–10]. Precise analysis of genetic screen data is particularly important given recent evidence that off-target effects can mislead targeted drug development efforts [11].

Previously, we developed an effective algorithm, the Bayesian Analysis of Gene Essentiality (BAGEL), for classifying essential and non-essential genes in pooled library gene perturbation screens using either CRISPR or shRNA [12, 13]. BAGEL calculates the log likelihood that a gene belongs to either the “essential” or the “non-essential” class, and returns a log Bayes Factor (BF) that, in the context of a typical genome-scale knockout screen in a cell line, represents a blend of statistical confidence and biological effect size. The classifier is trained using gold-standard reference sets of likely core-essential and non-essential genes, themselves derived from genetic screens and gene expression studies [12, 14]. Provided appropriate care is taken to prevent circularity, these gold standards also offer an unbiased yardstick against which to compare the performance of other algorithms, screening technologies, and experimental designs.

Despite its utility, the previous version of BAGEL has some notable limitations. Firstly, it used a truncated fold change model to calculate Bayes Factor, which capped the dynamic range of Bayes Factors. Secondly, though the bootstrapping approach it uses to train models is robust, it is computationally expensive, resulting in long run times under normal conditions. Lastly, there is no provision for correcting copy number amplification effects [5, 9, 15] or multi-targeting gRNA effects. To address these limitations, we have developed a new version of the software, BAGEL2 [16, 17], which we present here. While the core algorithm remains intact, we present several changes that improve the run time and accuracy of BAGEL, including a correction for gRNA off-target effects and an increased dynamic range of BFs that enables the detection of tumor suppressor genes whose knockout gives rise to increased cellular fitness. While BAGEL2 does not contain a method to address copy number artifacts, we describe a pipeline using CRISPRcleanR [18] for correcting these effects.

## Implementation

### BAGEL pipeline summary

BAGEL takes a tab-separated plain text file of gRNA read counts as input. We employed a third-party application, CRISPRcleanR [18], to calculate fold change with copy number effect correction. Alternatively, there is a built-in “fc” function in BAGEL application to calculate fold change if copy number correction is not desired. After that, essentiality was calculated using the BAGEL “bf” function from the fold change file. Finally, benchmarking by precision and recall of reference genes was conducted using the BAGEL “pr” function.

### Preparing a read count file

If a screen analysis starts from a fastq file of reads, alignment into reference sgRNA library can be conducted using Bowtie version 1.1.2. Since we expect no duplicate sgRNAs in the library, parameters -v 0 -m 1 to search reads with no mismatches (-v 0), and discarding reads which map to multiple index sequences (-m 1) was used for best accuracy. Then, read counts can be generated by parsing the resulting SAM file. An alternative pipeline is to use MAGeCK [19] to tabulate sgRNA reads.

### Calculate fold change from read count file and correct copy number effect using CRISPRcleanR

CRISPRcleanR was downloaded from github (https://github.com/francescojm/CRISPRcleanR). To run CRISPRcleanR, we built alignment information of a CRISPR library. We mapped positions and targeted exons of gRNAs using gencode annotation v28 for genome build GRCh37. Since CRISPRcleanR generates one summarized fold change for all input replicates, we ran CRISPRcleanR for each replicate separately. Then, we pasted them into one file as separate columns. Otherwise this, we ran CRISPRcleanR by the default practice provided by the author.

### Bayes Factor (BF) calculation—BAGEL2 “bf” function

The BAGEL2 “bf” function is a tool for calculating the log Bayes Factor (BF), which quantifies the degree of support for selecting one model over another (i.e., essential nor non-essential). The BF can be thought of as a combined metric of statistical significance and effect size [12]. The formula of Bayes Factor defined previously is as below:

$$ \mathrm{BF}=\frac{\Pr \left(D|\mathrm{essential}\right)}{\Pr \left(D|\mathrm{non}-\mathrm{essential}\right)}=\frac{\int \Pr \left(D|k,\mathrm{essential}\right)\Pr \left(k|\mathrm{essential}\right) dk}{\int \Pr \left(D|k,\mathrm{non}-\mathrm{essential}\right)\Pr \left(k|\mathrm{non}-\mathrm{essential}\right) dk} $$

**Fig. 1:**
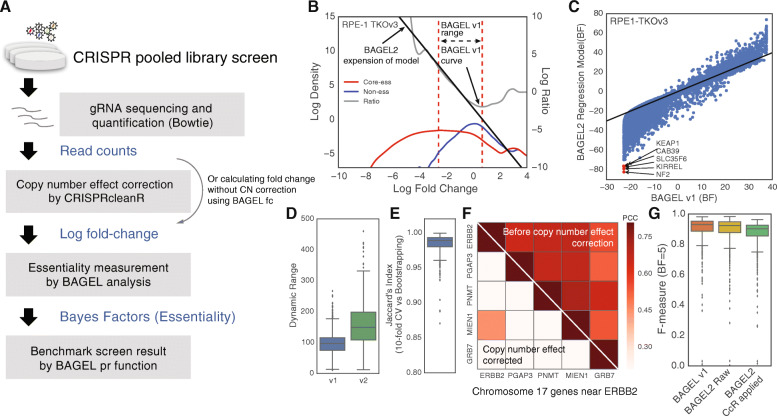
Improvement of BAGEL algorithm. **a** A brief flow diagram of CRISPR pooled library screen analysis using BAGEL pipeline. **b** Improvements in the model selection algorithm. The red and the blue curves indicate kernel density plots of fold changes of reference core-essential and non-essential genes, respectively. The gray curve indicates the ratio (difference in logs) of core-essential density to non-essential density at the point of fold change. Since there are few data points in the marginal area, BAGEL limited calculation area of fold change between the point that blue curve hits the density threshold (2^−7^ was used in BAGEL) as a lower bound and the first local minimum ratio as an upper bound (red dashed lines). In BAGEL2, we employed linear regression to interpolate marginal area outside this region (black line). **c** Comparison of gene essentiality (Bayes Factor) between BAGEL and BAGEL2 using RPE-1 cell line screened by TKOv3. Known tumor suppressors (*NF2*, *KIRREL*, and *KEAP1*) that are scored BF ~ − 20 with hundreds of other genes in BAGEL were measured as much lower Bayes Factor and distinguished clearly from others in BAGEL2. **d** Dynamic range of BAGEL2 results were increased from BAGEL across screens in the Avana dataset. **e** Jaccard index between predicted essential gene sets in Avana by 10-fold cross-validation and bootstrapping. **f** Pearson correlation coefficient of essentiality across 517 cell lines in Avana data between frequently amplified genes near *ERBB2* on chromosome 17. After CRISPRcleanR is applied, essentiality correlation due to copy number amplification effect was successfully corrected. **g** Prediction performance benchmark between BAGEL, BAGEL2 applied linear interpolation and 10-fold cross-validation (BAGEL2 Raw), and BAGEL2 + CRISPRcleanR applied version (BAGEL2 CCR applied)

To implement this, BAGEL2 resamples all genes in the dataset into training set and test set by either 10-fold cross-validation or bootstrapping, a user-selectable option. In each iteration of sampling, BAGEL2 uses kernel density estimation to generate fold change distributions for essential and non-essential models, using all guides targeting control essential or non-essential genes in the resample. A guide-level log BF is then estimated as the log-ratio of these two distributions, Pr(Ess)/Pr(Non) (Fig. 1b, gray curve). However, as described in Hart and Moffat [13], this log ratio is unstable outside the region of dense data for both distributions (Fig. 1b, red dashed lines). Where BAGEL truncates data to the stable region, BAGEL2 builds a linear regression model of log likelihood ratio within this region to extrapolate log ratios outside it (Fig. 1b). Moreover, whereas BAGEL used a hardcoded limit to set the truncation region, BAGEL2 employs a log decay function to calculate the thresholds, making BAGEL2 more useful for small CRISPR library screens (Additional file 1: Fig. S1). Core-essential (CEGv2) and non-essential (NEG) gene sets are defined in our previous studies [12, 13]. An sgRNA-level log Bayes Factor (hereafter, all BF are log BF) is calculated using this regression model, and replicate-level sgRNA BF is summed to a screen-level sgRNA BF. The gene-level Bayes Factor is calculated as the sum of sgRNA-level BF.

### Correcting multi-targeting effects

The dropout phenotype of a guide RNA is the sum of the effects from target gene knockout, locus-independent DNA cleavage, and off-target effects from other loci, possibly including non-additive genetic interactions. In an effort to remove guide-level effects that are independent of the targeted gene, we developed a multi-targeting correction algorithm. The algorithm estimates and removes the “incremental BF” induced by off-target DNA cleavage sites within one mismatch, while excluding the confounding effects of off-target gene knockout. For example, consider the case of a target gene A with four sgRNAs. Of these, gRNA1 targets multiple other protein-coding genes, gRNA2 targets gene A and off-target non-coding regions, and gRNA3-4 target only gene A (Additional file 1: Fig. S2). Then, the Bayes Factor of gRNA1 can be described as the sum of the BF from the loci it targets, plus potential interaction terms:

$$ {\displaystyle \begin{array}{c}\mathrm{BF}(gRNA1)=\left({g}_A+t\right){p}_{\mathrm{perfect}}\\ {}+\left({g}_B\times {w}_B+t\right){p}_{\mathrm{perfect}}+\left({g}_C\times {w}_C+t\right){p}_{\mathrm{perfect}}\\ {}\begin{array}{c}+\left({g}_D\times {w}_D+t\right){p}_{1 bp-\mathrm{mismatch}}\\ {}+\left({g}_D\times {w}_D+t\right){p}_{1 bp-\mathrm{mismatch}}\dots \\ {}\mathrm{where}\ g=\mathrm{effect}\ \mathrm{of}\ \mathrm{target}\ \mathrm{gene}\ \mathrm{knockout},\\ {}\begin{array}{c}{g}_A=\mathrm{On}\ \mathrm{target}\ \mathrm{gene},\\ {}{g}_B,{g}_C=\mathrm{Off}\ \mathrm{target}\ \mathrm{protein}\ \mathrm{coding}\ \mathrm{gene}\mathrm{s}\ \left(\mathrm{perfectmatch}\right)\\ {}{g}_D,{g}_E=\mathrm{Off}\ \mathrm{target}\mathrm{s}\ \mathrm{protein}\ \mathrm{coding}\ \mathrm{gene}\mathrm{s}\ \left(1\mathrm{bp}\ \mathrm{mismatch}\right).\\ {}\begin{array}{c}t=\mathrm{DNAcleavageeffect},\\ {}w=\mathrm{genetic}\ \mathrm{interaction}\ \mathrm{to}\ \mathrm{other}\ \mathrm{target}\ \mathrm{gene}\mathrm{s}\ \left(1=\mathrm{no}\ \mathrm{interaction}\right),\\ {}p=\mathrm{probabilityofcleavage}\end{array}\end{array}\end{array}\end{array}} $$

Since guides that target multiple protein-coding loci can induce off-target locus-specific effects, it is inappropriate to use gRNA1 to calculate a locus-independent DNA cleavage effect, and gRNA1 is discarded. To calculate the incremental BF, we only considered gRNAs targeting only gene A plus potential off-target non-coding regions. Thus, the BF of gRNA2-4 are:

$$ {\displaystyle \begin{array}{c}\mathrm{BF}(gRNA2)=\left({g}_A+t\right){p}_{\mathrm{perfect}}\\ {}+(t){p}_{\mathrm{perfect}}+(t){p}_{\mathrm{perfect}}\dots \\ {}\begin{array}{c}+(t){p}_{1 bp-\mathrm{mismatch}}+(t){p}_{1 bp-\mathrm{mismatch}}\dots \\ {}={g}_A+{n}_{\mathrm{perfect}}t{p}_{\mathrm{perfect}}+{n}_{1 bp-\mathrm{mismatch}}t{p}_{1 bp-\mathrm{mismatch}}\\ {}\mathrm{BF}\left( gRNA3,4\right)=\left({g}_A+t\right){p}_{\mathrm{perfect}}\end{array}\end{array}} $$

Then, the incremental BF between gRNA2 and the average of gRNA3 and gRNA4 is:

$$ \mathrm{BF}\ \mathrm{Increment}=\mathrm{BF}(gRNA2)-\mathrm{mean}\left(\mathrm{BF}\left( gRNA3,4\right)\right)=\left({n}_{\mathrm{perfect}}-1\right)t{p}_{\mathrm{perfect}}+{n}_{1 bp-\mathrm{mismatch}}t{p}_{1 bp-\mathrm{mismatch}} $$

**Fig. 2:**
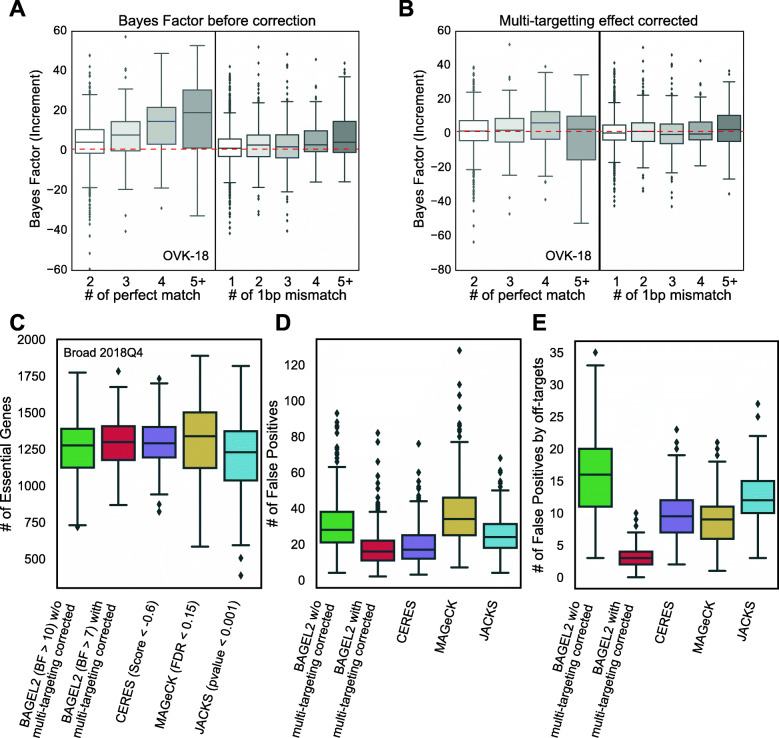
The multi-targeting effect correction reduces false positives from off-targets with 1-bp mismatch. **a**, **b** Increment of Bayes Factors of multi-targeting gRNAs but targeting only a single protein-coding gene in comparison with Bayes Factor of gRNAs targeting the protein-coding gene without any other targets **a** before the multi-targeting effect correction and **b** after the multi-targeting effect correction. **c** The number of essential genes across good quality cell lines (F-measure > 0.85) in the Avana dataset predicted by BAGEL2 with or without CRISPRcleanR and other algorithms, CERES, MAGeCK, and JACKS with cut-off threshold BF 10, BF 7, score − 0.6, FDR 0.15, and *p* value 0.001, respectively. The cut-off threshold was aimed for obtaining similar numbers of essential genes. **d** The number false positives predicted by each algorithm. False positives were defined by non-expressed genes in RNA-seq data of corresponding cell lines. BAGEL2 after multi-targeting effect correction shows comparable results with CERES and much lower numbers than results of MAGeCK and JACKS. **e** The number of false positives in predicted essential genesets when the scope is limited to genes having gRNAs mapped over than five 1-bp mismatched targets that are likely from multi-targeting effects of 1-bp mismatched targets. The result of BAGEL2 after correction shows the best performance among algorithms

Aggregating data across many genes, we can estimate *tp*_perfect_ and *tp*_1*bp* − mismatch_ by multiple linear regression (Fig. 2a, b, and Additional file 1: Fig. S2). Finally, we applied a guide-level BF penalty based on the number of off-target perfect match and single-mismatch sites:

$$ {\displaystyle \begin{array}{c}\mathrm{B}{\mathrm{F}}^{\prime }=\mathrm{BF}-\left[\left({n}_{\mathrm{perfect}}-1\right){tp}_{\mathrm{perfect}}+{n}_{1 bp-\mathrm{mismatch}}t{p}_{1 bp-\mathrm{mismatch}}\right]\\ {} tp=\mathrm{estimated}\ \mathrm{by}\ \mathrm{multiple}\ \mathrm{linear}\ \mathrm{regression}\ \mathrm{model}\end{array}} $$

### F-measure and false discovery rate (FDR) calculation

F-measure or *F*_1_ score (BF = 5) is the harmonic mean of precision and recall at the threshold of Bayes Factor 5 and it can represent the performance of essentiality prediction. We calculate F-measure using a precision-recall table generated by BAGEL pr function.

$$ \mathrm{Precision}=\frac{\mathrm{TP}}{\mathrm{TP}+\mathrm{FP}} $$

$$ \mathrm{Recall}=\frac{\mathrm{TP}}{\mathrm{TP}+\mathrm{FN}} $$

TP = positives in reference+ core-essential set

FP = positives in reference non-essential set

FN = negatives in reference core-essential set

Since it is rare that a gene is exactly BF 5, we used the precision and the recall that is the nearest but greater than BF 5. False discovery rate used in this study was calculated as 1.0 – Precision.

### Acquiring publicly available screen data

There are large-scale, public CRISPR screen datasets for cancer cell lines such as Depmap (Avana dataset) by Broad Institute [5] and Project Score by Sanger Institute (Score dataset) [6]. We downloaded read count data for the Avana 2018Q4 release, which contains screens of 517 cancer cell lines, from the DepMap official website (http://www.depmap.org). Since the Avana library contains sgRNA targeting genetic loci, we discarded gRNAs targeting multiple protein-coding genes without mismatch at the read count-level data based on the guide-gene map of the Avana library. Protein-coding gene information was obtained from CCDS [20] (06.14.2018 version, genes annotated as Public or Reviewed, update pending). We also downloaded read count data of Project Score for 339 cancer cell lines from the official website (https://depmap.sanger.ac.uk/). Gene names used in read counts were updated based on NCBI official symbols. Then, we applied standard BAGEL2 pipeline with CRISPRcleanR copy number effect correction [16]. Since DepMap screens were conducted in four different batches, we used corresponding pDNA read counts as controls of each batch number. We used pDNA Avana4_010115_1.5Ex_batch0 and pDNA Avana4_060115_1.5Ex_batch0 for batch 0 screens; pDNA Avana4_010115_1.5Ex_batch1, pDNA Avana4_060115_1.5Ex_batch1, “pDNA Avana4_0101215_0.55Ex_batch1,” and pDNA Avana4_060115_0.55Ex_batch1 for batch 1 screens; Avana4pDNA20160601-311cas9 RepG09_batch2, Avana4pDNA20160601-311cas9 RepG10_batch2, Avana4pDNA20160601-311cas9 RepG11_batch2, and Avana4pDNA20160601-311cas9 RepG12_batch2 for batch 2 screens; and Avana 4+ Hu pDNA (M-AA40, 9/30/15)_batch3, Avana 4+ Hu pDNA (M-AA40, 9/30/15) (0.2 pg/uL)_batch3, and Avana 4+ Hu pDNA (M-AA40, 9/30/15) 0.2 pg/uL_batch3 for batch 3 screens. For Project Score screen analysis, we used “ERS717283.plasmid” as a control of screens. For RPE-1 cells, we re-analyzed screens used in Hart et al. [14]. Processed Bayes Factor tables are downloadable on Figshare [17].

### Essentiality calculation using other dependency identifiers, MAGeCK, JACKS, and CERES

We downloaded MAGeCK [19] version 0.5.9.3 from the MAGeCK distribution website (https://sourceforge.net/p/mageck/wiki/Home/) and applied it to Avana read count data with default parameters. For CERES [5], we used pre-calculated 2018Q4 dependency data downloaded from the Depmap official website. We also downloaded JACKS [21] from the official github page (https://github.com/felicityallen/JACKS) and ran it for the Avana read count data with gene guide map and replicate information. To decide whether a gene is essential or not, we used “neg|fdr” for MAGeCK, dependency score for CERES, and *p* value for JACKS.

### False positive analysis

To estimate screen false positives, we downloaded CCLE RNA-seq log TPM data for approx. 1000 cell lines from DepMap [22]. False positives of each cell line were defined log-expression below 1.0. Since the number of false positives was sensitive to the number of essential genes, we used varying thresholds to keep the number of essential genes similar across pipelines (Fig. 2c). The thresholds were BF > 10 for BAGEL2 data without correction and BF > 7 for BAGEL2 data with multi-targeting correction. Additional thresholds were score − 0.6, FDR 0.15, and *p* value 0.001 for CERES, MAGeCK, and JACKS, respectively. Then, we counted how many false positives were present in essential gene calls from each algorithm across good quality cell lines (F-measure > 0.85) in the Avana dataset. For an alternative definition of false positives, we downloaded shRNA screens analyzed by DEMETER2 [23] from DepMap and defined genes above score zero as non-essential genes of each cell line.

### Quality score analysis

Measuring the quality of a single replicate is important to inform experimental strategy. We measured Cohen’s *D* of reference core-essential genes and non-essential genes as a quality score of single replicates.

$$ \mathrm{Quality}\ \mathrm{Score}=\mathrm{Cohe}{\mathrm{n}}^{\prime}\mathrm{s}\ D=\frac{\mathrm{mean}\ {fc}_{\mathrm{n}\mathrm{on}-\mathrm{essential}}-\mathrm{mean}\ {fc}_{\mathrm{core}-\mathrm{essential}}\ }{\mathrm{pooled}\ \mathrm{standard}\ \mathrm{deviation}} $$

For each single replicate in the Avana dataset, we collected all log fold change values of gRNAs targeting either reference core-essential genes or non-essential genes. To demonstrate the relationship between prediction performance and quality of single-replicate screens, we compared F-measure (BF = 5) from Bayes Factor using all replicates and mean quality score of each replicates calculated from fold change level.

## Results

### An improved log likelihood/regression model

The analysis pipeline for a loss-of-function fitness screen consists of three steps: (1) mapping reads to the guide sequences in the CRISPR library and building a table of read counts, (2) normalizing counts across samples and calculating guide-level fold change, and (3) compiling guide-level information into gene-level fitness scores (Fig. 1a). CRISPR screen analysis starts from the step of mapping raw sequencing read files to their corresponding CRISPR library. Mapping reads can be done with a variety of sequence analysis tools, including Bowtie [24], MAGeCK [19], and poolQ (https://portals.broadinstitute.org/gpp/public/software/poolq). Fold change is calculated by comparing endpoint to starting plasmid or T0 sample, a function now available in BAGEL2 using the *fc* option.

To calculate a gene essentiality score, BAGEL2 adopts the same Bayesian model selection approach as BAGEL. The “essential” model is represented by a kernel density estimate (KDE) of the distribution of guide-level fold changes of gRNA targeting a training set of essential genes [14], and the “non-essential” model is likewise trained on a set of non-essential genes (Fig. 1b) [12, 14]. Then, for each gRNA targeting each gene, a guide-level log Bayes Factor (BF) is calculated as the log ratio of these two kernel density estimates, evaluated at the observed log fold change of the guide.

The stability of this calculation depends heavily on the local density of data points used to calculate the training set KDEs. For example, at extreme fold changes, sparsity of training data from the non-essential set results in extreme ratios. For this reason, in the previous version of BAGEL, we defined the boundaries of the near-linear range of this ratio and truncated all data outside these boundaries (Fig. 1b). Guide-level log BFs are then summed to gene-level log BFs (hereafter all Bayes Factors are in log2 space). BAGEL2 relaxes this limitation by calculating a linear best fit to the log ratio in this space and using this fit to extrapolate the BF calculation to all observed fold changes (Fig. 1b, gray line). The net result is a better usage of the total fold change data, a correction for log-ratio instability at positive or extreme negative guide-level fold changes, and a broader dynamic range of gene-level BFs reported by the algorithm.

An unanticipated result of this broader dynamic range is that BAGEL2 now detects putative tumor suppressor genes. We re-analyzed a previously reported genome-scale screen of RPE1 retinal pigmented epithelium cells performed with the TKOv3 library [14], and comparing BAGEL to BAGEL2 results shows the truncation of gene-level BFs in BAGEL (Fig. 1c). Notably, outliers with extreme negative BFs in BAGEL2 (Fig. 1c, red) include genes with known tumor suppressor activity, including *KEAP1* [25, 26] and Hippo pathway genes *NF2* and *KIRREL* [27]. We confirmed the regression scheme increases dynamic range across hundreds of cell lines in the Avana dataset downloaded from DepMap [5] (Fig. 1d).

Another improvement in BAGEL2 involves replacing bootstrapping with 10-fold cross-validation. Bootstrap resampling of the training sets, used in BAGEL, provides a robust method to evaluate the effect of training data variance on gene-level BF calculations, but is computationally expensive. However, given the large size of training sets used for genome-scale fitness screens, resampling introduces relatively little variance. Ten-fold cross-validation yields nearly identical Bayes Factor distributions as bootstrapping in most cases, and comparing BAGEL and BAGEL2 hits (BF ≥ 5) in the DepMap data yields Jaccard coefficients ~ 0.99 (Fig. 1e). Cross-validation is the default setting in BAGEL2 and speeds up running time on a single processor by roughly 50-fold.

Copy number amplifications are a known source of potential artifacts in CRISPR knockout fitness screens [9, 15, 28], and BAGEL2 does not correct for this source of error. Instead, we employed an unsupervised copy number correction algorithm, CRISPRcleanR [18], as a preprocessing step. CRISPRcleanR corrects amplicon-induced artifacts based on guide position and fold change, without copy number information. We find that BAGEL2 with copy number correction preprocessing successfully reduces amplicon-induced artifacts (Fig. 1f) while maintaining high sensitivity and specificity (Fig. 1g). Overall, BAGEL2 improves performance and sensitivity over BAGEL.

### Correcting multi-targeting effects and false positive analysis

It is widely accepted that the specificity and sensitivity of CRISPR reagents far exceeds that of prior-generation shRNA reagents [4]. However, off-target effects of CRISPR reagents can still confound loss-of-function screens. Recently, several studies reported that CRISPR/Cas9 reagents have a non-negligible effect on off-target cut sites with mismatches of 1–2 bp from the intended target site [29, 30]. These off-targeting effects by mismatched targets can cause additional ad hoc DNA cutting or, depending on the locus, knockout of genes. We found that many of guide RNAs in the Avana and KY libraries target several sites with perfect matches, and our TKOv3 library was specifically designed to allow up to one perfect-match, off-target cut site in an intergenic region [14] (Additional file 1: Fig. S2). These multi-targeting gRNAs can result in unexpected fitness defects, the effect of which can be decomposed into target-specific and off-target/multiple-targeting effects (see Implementation). To implement multi-targeting effect correction in a single cell line screen, we re-aligned gRNA sequences of CRISPR/Cas9 libraries to the human genome with mismatches allowed. In this study, we only consider perfect matched targets and 1-bp mismatched targets. Using this alignment information, BAGEL discards promiscuous gRNAs (perfect match > 10 loci or 1-bp mismatch > 10 loci) from libraries. Then, to measure the component of fitness defect specific to multi-targeting effects, we took sgRNAs targeting multiple loci with 0–1 mismatch to non-protein-coding regions (excluding protein-coding off-target sites to minimize the contribution from other genes and genetic interactions; Additional file 1: Fig. S2). The multiple-targeting effects of gRNAs can be estimated by the incremental BF in comparison with gRNAs targeting the same gene but with no off-target cut sites. For example, in ovarian endometrioid cancer cell line OVK-18, the multiple-targeting effects of the Avana library showed an incremental BF due to off-targets that increased roughly linearly with the number of perfect-match, off-target cut sites in the genome and a smaller incremental guide-level BF with the frequency of mismatched off-target sites (Fig. 2a; Additional file 1: Fig. S2B). Since we only addressed sgRNAs targeting multiple loci but targeting only one protein-coding locus, these effects were exclusively from multi-targeting effects, not the effect of genetic interaction. In our example case, each additional perfect-match target boosted the BF of a single gRNA by 3.5 and each additional 1-bp mismatched target increased the BF by 1.4 (~ 40% of the boost attributable to perfect matches). We removed these off-target effects by guide-level regression of incremental BF vs. off-target effects (i.e., applied a BF penalty based on the number of predicted off-target cut sites) and confirmed that the bias was no longer present after the effect was removed (Fig. 2b).

We compared BAGEL2 with multi-targeting correction to BAGEL2 without correction, as well as to other contemporary screen analysis algorithms, including CERES [5], MAGeCK [19], and JACKS [21], run against the DepMap 2018Q4 data release, using raw read counts per guide as a starting point. Since the number of false positives is sensitive to the number of essential genes, we identified thresholds for each algorithm that returned roughly the same median number of essential genes across the 518 cell line screens analyzed (Fig. 2c). Then, for each cell line, we identified a set of genes with no to low expression (logTPM < 1), judging that genes with trace mRNA expression levels cannot be essential and following the concept used to define the non-essential reference gene set [12]. For each algorithm, we identified the total number of expression-defined false positives (Fig. 2d). BAGEL2 results after multi-targeting effect correction showed the lowest number of false positives and resulted in significantly fewer false positives than MAGeCK and JACKS, while CERES showed a similar number as BAGEL2. To further investigate whether the correction can minimize false positives from multi-targeting effects, we limited the scope to non-expressed genes targeted by gRNA with 5 or more 1-bp mismatched off-target cut sites in the genome. BAGEL2 multi-target correction effectively filters these genes (Fig. 2e). We also see consistent performance when using alternative definitions of false positives, including non-essential genes in matched shRNA screens (score > 0, DEMETER2 [23]) and reference non-essential genes (Additional file 1: Fig. S3). We further demonstrate that agreement of gene essentiality across cell lines screened using both the Avana and KY libraries can be improved by multi-targeting effect correction (Additional file 1: Fig. S3). Overall, we show that BAGEL2 can correct the multi-targeting effects from perfect-matched and 1-bp mismatched targets, reducing the number of false positives arising from promiscuous sgRNA effects, and that BAGEL2 accurately discriminates essential genes from non-essentials in comparison with other algorithms.

### Replicate quality score can predict performance of cell line screens

**Fig. 3:**
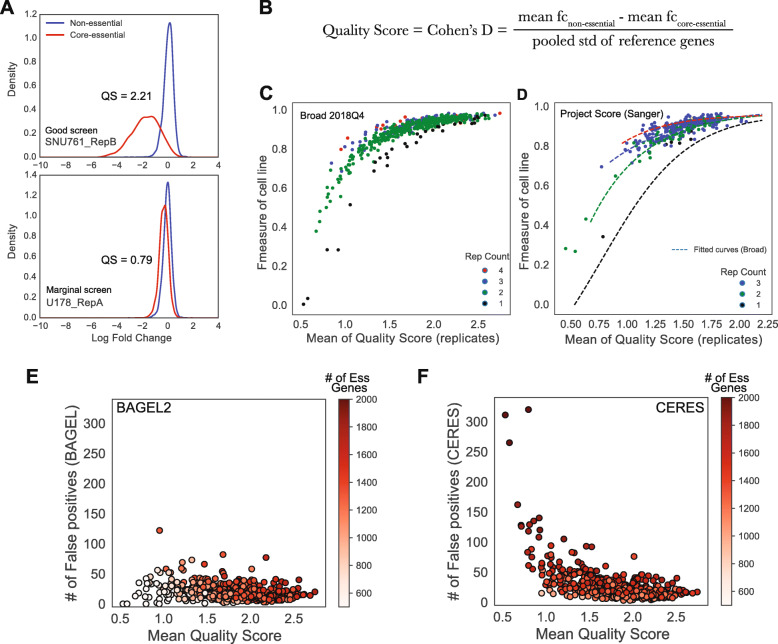
Variable screen performance. **a** Kernel density estimates of reference core-essential genes (red) and non-essential genes (blue) with SNU761 replicate B as an example of good screen (upper panel) and U178 replicate A as an example of marginal screen (lower panel). The good screen shows clear separation between core-essential and non-essential curves whereas the marginal screen shows less separation. **b** The equation of quality score. **c** Mean quality score of replicates and F-measure in cell line level shows clear correlation trends and differentiated by replicate screen counts per cell line. **d** Project Score CRISPR screen data recapitulated and followed the same trends of Avana set. **e**, **f** Relationship between the number of false positives in **e** BAGEL2 results and **f** CERES results across 517 cell lines in Avana data. Each dot colored by the number of essential genes

CRISPR screens require significant technical expertise, but even in the best hands, results can vary for numerous reasons, including environmental, experimental, and intrinsic factors such as variable Cas endonuclease efficiency, batch effects, PCR noise, stochastic off-target events of guide RNAs, and characteristics of individual cell lines [30–33]. Understanding and identifying effective and ineffective screens is necessary to understanding gene essentiality and differential essentiality. Previously, we defined lists of core-essential and non-essential genes [12, 14]. These reference gene sets are not only used as training sets in BAGEL, but also can be used to evaluate the quality of a screen (Fig. 3a). We compared a good screen (SNU-761, replicate B) and a marginal screen (U-178, replicate A) from the same batch in the Avana dataset. While the good screen shows clear separation of core-essential (red) and non-essential guides (blue), the marginal screen shows much greater overlap between the two distributions. To distinguish good from marginal screens, we employed as a quality score the Cohen’s *D* statistic, which is the difference in mean log fold change between core-essential and non-essential genes divided by the pooled standard deviation of log fold change of all reference genes (Fig. 3b). Using this scheme, we calculated the quality scores of SNU-761 (rep B) and U-178 (rep A) as 2.21 and 0.79, respectively (Fig. 3a). We further applied this quality score measurement to all individual replicates in the Avana dataset, and we compared with the F-measure of cell lines derived by BAGEL2 aggregation of all replicates (Fig. 3c). Many low-performing cell lines (F-measure < 0.7) included replicates having low mean quality score (below 1.0). Notably, even when average replicate quality is less than ideal, multiple replicates can boost overall screen quality; likewise, even high-quality, single-replicate screens show lower overall F-measure than equivalent quality screens with two or more replicates. We confirmed the generality of these trends by conducting the same analysis with data from Project Score [6] (Fig. 3d). The relationship between replicate quality score, number of replicates per screen, and overall screen F-measure was highly consistent with the Broad data and supports the applicability of the Cohen’s *D* statistic as a replicate-level quality score.

Although quality scores of replicates were directly related to the overall reliability of an experiment, we evaluated the effect of including one low-quality replicate in an otherwise high-quality screen. We took one replicate from a screen and added random noise to the fold change for each sgRNA, dropping the Cohen’s *D* from > 2 to ~ 1.1 (Additional file 1: Fig. S4). The overall trend supports the general notion that additional replicates can smooth out random noise and increase overall screen performance (Fig. 3c, d), and we find that a single low-quality replicate (quality score = 1.08) among one or more high confidence replicates (quality score > 2.0) has only marginal overall effect on performance (Additional file 1: Fig. S4).

### Data quality has different effects on different algorithms

Although BAGEL2 is robust to variation in data quality within a screen, overall screen quality can have profound effects on the results of an analytical pipeline. We compared how quality score affects the results of BAGEL2 and CERES (Fig. 3e, f, Additional file 2: Table S1). Interestingly, the two algorithms show opposite behavior as the quality of the underlying data degrades. In BAGEL2, the number of false positives remained similar across all quality levels, but BAGEL2 calls very few essential genes for low-quality data (Fig. 3e). In contrast, CERES amplifies the number of hits and the corresponding number of false positives as quality degrades (Fig. 3f). These results reflect the approaches adopted by the two algorithms. The Bayes Factor approach provides a summary statistic that essentially combines effect size and statistical significance. Since lower quality screens offer both lower effect size (fold change) and corresponding statistical power, the number of essential genes in a lower quality screen will be fewer than in a high-quality screen. In contrast, CERES rescales results by setting the score of core-essential genes to − 1.0 and non-essential genes to zero. Since low-quality screens poorly distinguish between essential genes and non-essential genes (Fig. 3a), significant error can be introduced by this rescaling. It should be noted that most CRISPR data in the DepMap and Project Score are of sufficiently high quality that this is not an important factor (95% of screens have quality scores > 1.0); nevertheless, researchers should be wary when including marginal quality screens in their analyses.

## Conclusions

In this study, we introduced an improved version of BAGEL algorithm, BAGEL2, for genome-wide pooled library loss-of-function fitness screens. We showed the linear interpolation of score expands the dynamic range of Bayes Factor in comparison to the previous version of BAGEL, enabling more accurate quantitation of fitness defects as well as discovery of putative tumor suppressor genes whose knockout results in faster proliferation.

We show that BAGEL2 can remedy false positives caused by CRISPR multi-targeting guides. That these effects can be mitigated algorithmically is important and useful. However, in the future, this effect should be addressed at the library design level. In particular, the Avana library contains many multi-targeting sgRNAs, compared to the Brunello [34], TKOv3 [14], and KY libraries [6]. However, there is clearly an advantage to screening with the Avana library and comparing results with the large and growing corpus of cell line characterization data available. Researchers will have to make their own informed decisions weighing these advantages and disadvantages.

To correct false positives caused by copy number amplification, we employed an unsupervised correction algorithm, CRISPRcleanR, in the BAGEL pipeline. Correlation analysis of genes in amplified regions demonstrated CRISPRcleanR corrected unexpected depletion adequately. Since CRISPRcleanR does not require copy number information for correction, it has an advantage for screens that do not have accompanying copy number data such as PDX models. However, in rare cases, we noticed CRISPRcleanR falsely corrected regions of high density of essential genes. Therefore, if copy number information is available, other supervised algorithms such as Crispy [15] may yield better results.

We suggest the Cohen’s *D* statistic, evaluated against reference core-essential and non-essential genes, to provide a quantitative measure of the quality of single screen replicates. We show that, as expected, the number and quality of replicates is directly related to the overall screen performance (F-measure). Interestingly, however, we also show that individual “bad” replicates do not seriously degrade the overall performance of an otherwise “good” screen. Nevertheless, we recommend evaluating quality at the replicate level and, if performance suffers, discarding low-quality outliers from groups of otherwise high-quality replicates.

## Availability and requirements

Project name: BAGEL2

Project home page: https://github.com/hart-lab/bagel

Operating system(s): Platform independent

Programming language: Python3

Other requirements: scipy, pandas, numpy, click, sklearn

License: MIT license

Any restrictions to use by non-academics: No restriction for non-academic use

## Supplementary Information

**Additional file 1: Fig. S1.** A) A brief flow diagram of CRISPR pooled library screen analysis using BAGEL pipeline with additional description about threshold B) A brief scheme of downsampling analysis for defining a log decay threshold function C) A scatter plot represented log-density after down-sampling (50%, 25%, 10%, 5%, 1%) at the original left side x limit, X_L0_. D) A plot of maximum log density of each down-sampling proportion using DepMap screens and the applied log-decay function in BAGEL2 derived from DepMap Achilles CRISPR screens. **Fig. S2.** A) A plot explains how to calculate increment of Bayes Factor for measuring multi-targeting effect. B) A two-dimensional dot plot gRNAs targeting multiple regions but only targeting one protein-coding gene. Each dot is located at the number of perfect-matched targets and 1-bp mismatched targets with random jitter and colored by increment of Bayes Factor. **Fig. S3.** A,B) The number of false positives defined by A) non-essential genes in matched shRNA screens (score > 0, DEMETER2) and B) reference non-essential genes in predicted essential genesets when the scope is limited to genes having gRNAs mapped over than five 1-bp mismatched targets that are likely from multi-targeting effects of 1-bp mismatched targets. C,D) Agreement of genes that have gRNAs targeting 5 or more regions with 1-bp mismatch between Sanger data (Project Score data) and Broad data (Avana dataset) A) before multi-targeting effect correction and B) after multi-targeting effect correction. **Fig. S4.** A,B) Fold change distribution plots for a replicate of HUP-T3 cell (A) before and (B) after fold change perturbation. To generate an low performance outlier sample, we added random noise to foldchange value. C) Quality scores of each replicate. D) F-measures (BF = 5) of combination of replicates. Adding the outlier (replicate D) to other high confident replicates reduce overall performance in condition of a few replicates (A vs AD and AB vs ABD).

**Additional file 2: Table S1.** The number of dependency calls in BAGEL2 (BF > 7) and CERES (score < − 0.6) and false positives in the calls across DepMap 2018Q4 screens with expression data (515 cells).

## Data Availability

All software described in this manuscript, as well as all processed data files used for analysis, are available (under the MIT license) at the Hart Lab github site and figshare [16, 17]: https://github.com/hart-lab/bagel https://figshare.com/projects/BAGEL2_Figshare/80690 All raw CRISPR fitness screen data used in this study can be downloaded from the DepMap database [5], the Project Score database [6], and Hart et al. [14]. CCLE RNA-seq data [22] used in this study is downloadable from the DepMap database.

## References

[1] Zhou Y, Zhu S, Cai C, Yuan P, Li C, Huang Y (2014). High-throughput screening of a CRISPR/Cas9 library for functional genomics in human cells. Nature..

[2] Shalem O, Sanjana NE, Hartenian E, Shi X, Scott DA, Mikkelsen TS (2014). Genome-scale CRISPR-Cas9 knockout screening in human cells. Science..

[3] Hart T, Chandrashekhar M, Aregger M, Steinhart Z, Brown KR, MacLeod G (2015). High-resolution CRISPR screens reveal fitness genes and genotype-specific cancer liabilities. Cell..

[4] Evers B, Jastrzebski K, Heijmans JPM, Grernrum W, Beijersbergen RL, Bernards R (2016). CRISPR knockout screening outperforms shRNA and CRISPRi in identifying essential genes. Nat Biotechnol.

[5] Meyers RM, Bryan JG, McFarland JM, Weir BA, Sizemore AE, Xu H (2017). Computational correction of copy number effect improves specificity of CRISPR-Cas9 essentiality screens in cancer cells. Nat Genet.

[6] Behan FM, Iorio F, Picco G, Gonçalves E, Beaver CM, Migliardi G (2019). Prioritization of cancer therapeutic targets using CRISPR-Cas9 screens. Nature..

[7] Wang T, Birsoy K, Hughes NW, Krupczak KM, Post Y, Wei JJ (2015). Identification and characterization of essential genes in the human genome. Science..

[8] Wang T, Yu H, Hughes NW, Liu B, Kendirli A, Klein K (2017). Gene essentiality profiling reveals gene networks and synthetic lethal interactions with oncogenic Ras. Cell.

[9] Aguirre AJ, Meyers RM, Weir BA, Vazquez F, Zhang C-Z, Ben-David U (2016). Genomic copy number dictates a gene-independent cell response to CRISPR/Cas9 targeting. Cancer Discov.

[10] Steinhart Z, Pavlovic Z, Chandrashekhar M, Hart T, Wang X, Zhang X (2017). Genome-wide CRISPR screens reveal a Wnt-FZD5 signaling circuit as a druggable vulnerability of RNF43-mutant pancreatic tumors. Nat Med.

[11] Lin A, Giuliano CJ, Palladino A, John KM, Abramowicz C, Yuan ML, et al. Off-target toxicity is a common mechanism of action of cancer drugs undergoing clinical trials. Sci Transl Med. 2019;11:eaaw8412. 10.1126/scitranslmed.aaw8412 PMC7717492 31511426

[12] Hart T, Brown KR, Sircoulomb F, Rottapel R, Moffat J (2014). Measuring error rates in genomic perturbation screens: gold standards for human functional genomics. Mol Syst Biol.

[13] Hart T, Moffat J (2016). BAGEL: a computational framework for identifying essential genes from pooled library screens. BMC Bioinformatics.

[14] Hart T, Tong AHY, Chan K, Van Leeuwen J, Seetharaman A, Aregger M (2017). Evaluation and design of genome-wide CRISPR/SpCas9 knockout screens. G3 (Bethesda).

[15] Gonçalves E, Behan FM, Louzada S, Arnol D, Stronach EA, Yang F (2019). Structural rearrangements generate cell-specific, gene-independent CRISPR-Cas9 loss of fitness effects. Genome Biol.

[16] Kim E, Hart T. BAGEL2 software. Hart Lab; 2020 [cited 2020 Oct 29]. Available from: https://github.com/hart-lab/bagel.

[17] Kim E, Hart T. BAGEL2 Figshare. figshare. [cited 2020 Oct 29]. Available from: https://figshare.com/projects/BAGEL2_Figshare/80690.

[18] Iorio F, Behan FM, Gonçalves E, Bhosle SG, Chen E, Shepherd R (2018). Unsupervised correction of gene-independent cell responses to CRISPR-Cas9 targeting. BMC Genomics.

[19] Li W, Xu H, Xiao T, Cong L, Love MI, Zhang F (2014). MAGeCK enables robust identification of essential genes from genome-scale CRISPR/Cas9 knockout screens. Genome Biol.

[20] Pujar S, O’Leary NA, Farrell CM, Loveland JE, Mudge JM, Wallin C (2018). Consensus coding sequence (CCDS) database: a standardized set of human and mouse protein-coding regions supported by expert curation. Nucleic Acids Res.

[21] Allen F, Behan F, Khodak A, Iorio F, Yusa K, Garnett M (2019). JACKS: joint analysis of CRISPR/Cas9 knockout screens. Genome Res.

[22] Ghandi M, Huang FW, Jané-Valbuena J, Kryukov GV, Lo CC, McDonald ER (2019). Next-generation characterization of the Cancer Cell Line Encyclopedia. Nature..

[23] McFarland JM, Ho ZV, Kugener G, Dempster JM, Montgomery PG, Bryan JG (2018). Improved estimation of cancer dependencies from large-scale RNAi screens using model-based normalization and data integration. Nat Commun.

[24] Langmead B, Trapnell C, Pop M, Salzberg SL (2009). Ultrafast and memory-efficient alignment of short DNA sequences to the human genome. Genome Biol.

[25] Ohta T, Iijima K, Miyamoto M, Nakahara I, Tanaka H, Ohtsuji M (2008). Loss of Keap1 function activates Nrf2 and provides advantages for lung cancer cell growth. Cancer Res.

[26] Taguchi K, Yamamoto M (2017). The KEAP1-NRF2 system in cancer. Front Oncol.

[27] Couzens AL, Knight JDR, Kean MJ, Teo G, Weiss A, Dunham WH (2013). Protein interaction network of the mammalian Hippo pathway reveals mechanisms of kinase-phosphatase interactions. Sci Signal.

[28] Munoz DM, Cassiani PJ, Li L, Billy E, Korn JM, Jones MD (2016). CRISPR screens provide a comprehensive assessment of cancer vulnerabilities but generate false-positive hits for highly amplified genomic regions. Cancer Discov..

[29] Fortin J-P, Tan J, Gascoigne KE, Haverty PM, Forrest WF, Costa MR (2019). Multiple-gene targeting and mismatch tolerance can confound analysis of genome-wide pooled CRISPR screens. Genome Biol.

[30] Wienert B, Wyman SK, Richardson CD, Yeh CD, Akcakaya P, Porritt MJ (2019). Unbiased detection of CRISPR off-targets in vivo using DISCOVER-Seq. Science..

[31] Lahens NF, Kavakli IH, Zhang R, Hayer K, Black MB, Dueck H (2014). IVT-seq reveals extreme bias in RNA sequencing. Genome Biol.

[32] Parekh S, Ziegenhain C, Vieth B, Enard W, Hellmann I (2016). The impact of amplification on differential expression analyses by RNA-seq. Sci Rep.

[33] Dempster JM, Pacini C, Pantel S, Behan FM, Green T, Krill-Burger J (2019). Agreement between two large pan-cancer CRISPR-Cas9 gene dependency data sets. Nat Commun.

[34] Sanson KR, Hanna RE, Hegde M, Donovan KF, Strand C, Sullender ME (2018). Optimized libraries for CRISPR-Cas9 genetic screens with multiple modalities. Nat Commun.

