# Supplementary material for: Improved analysis of CRISPR fitness screens and reduced off-target effects with the BAGEL2 gene essentiality classifier
Source: Genome Med. 2021 Jan 6;13:2. doi: 10.1186/s13073-020-00809-3 (PMC7789424; doi:10.1186/s13073-020-00809-3)

Fig S1. A) A brief flow diagram of CRISPR pooled library screen analysis using BAGEL pipeline with additional description about threshold B) A brief scheme of downsampling analysis for defining a log decay threshold function C) A scatter plot represented log-density after down-sampling (50%, 25%, 10%, 5%, 1%) at the original left side x limit, XL0. D) A plot of maximum log density of each down-sampling proportion using DepMap screens and the applied log-decay function in BAGEL2 derived from DepMap Achilles CRISPR screens.

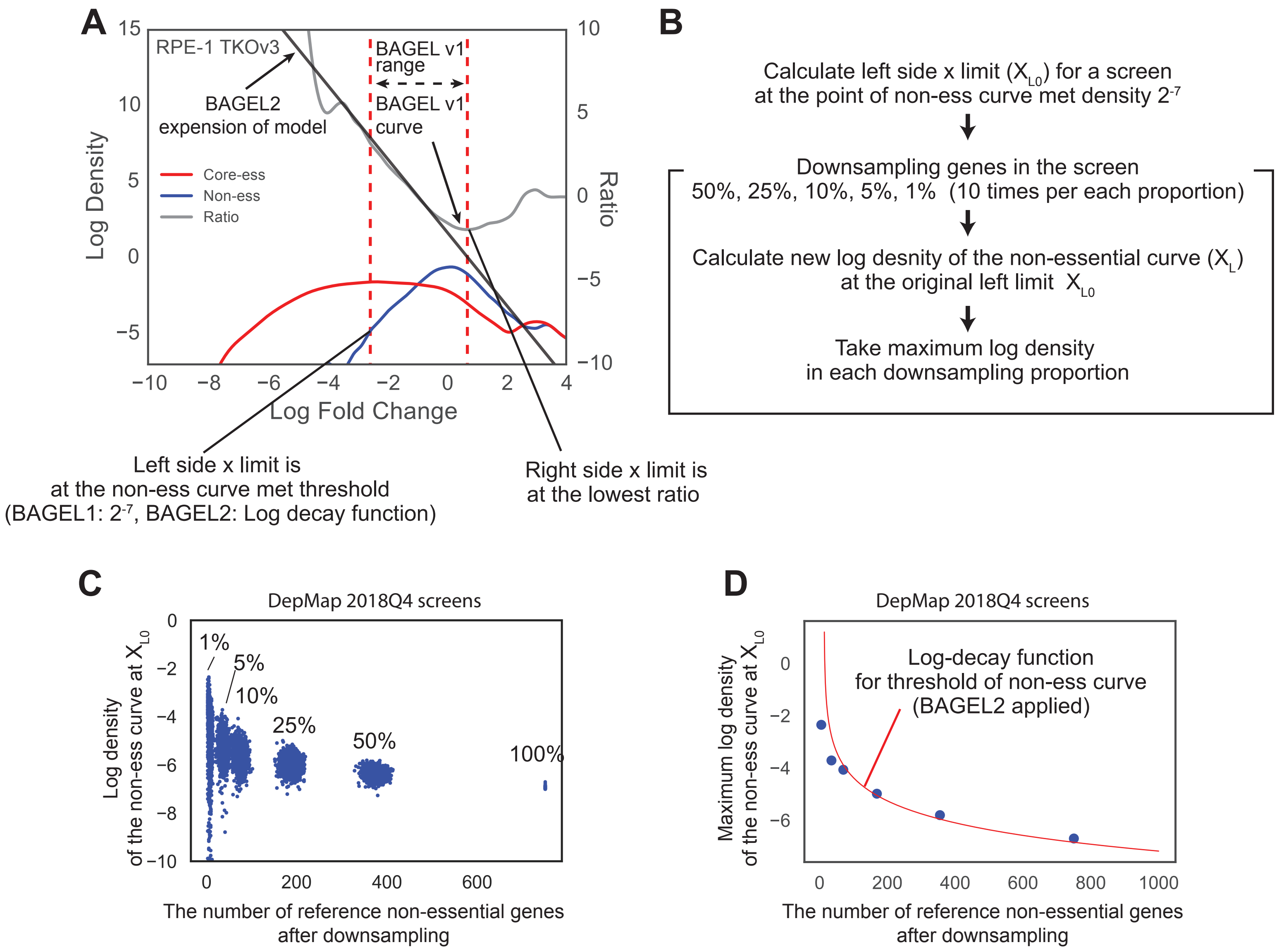

Fig S2. A) A plot explains how to calculate increment of Bayes Factor for measuring multi-targeting effect. B) A two-dimensional dot plot gRNAs targeting multiple regions but only targeting one protein-coding gene. Each dot is located at the number of perfect-matched targets and 1-bp mismatched targets with random jitter and colored by increment of Bayes Factor.

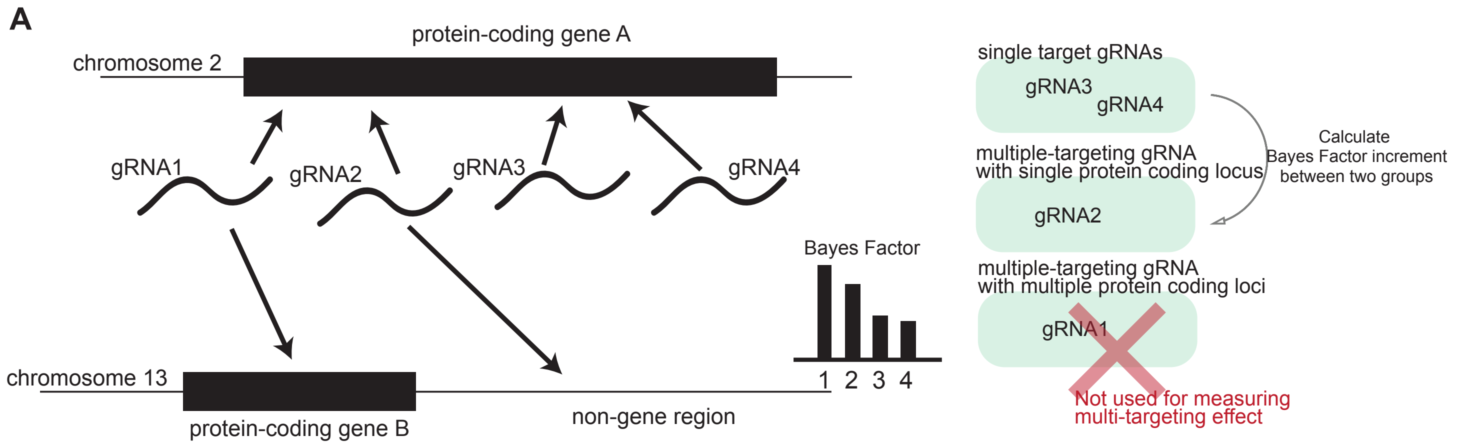

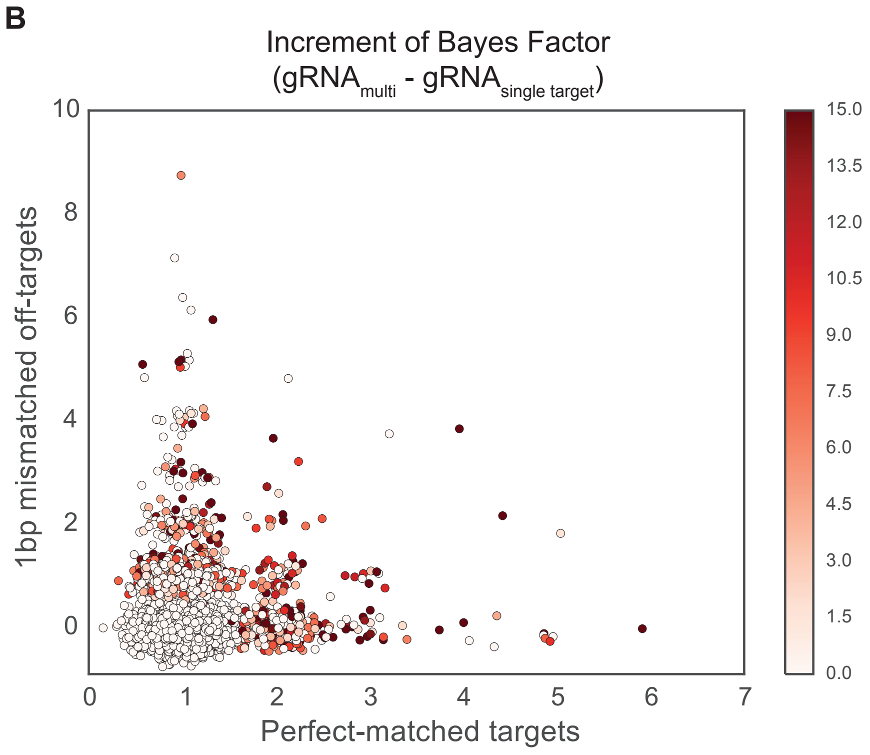

Fig S3. A,B) The number of false positives defined by A) non-essential genes in matched shRNA screens (score > 0, DEMETER2) and B) reference non-essential genes in predicted essential genesets when the scope is limited to genes having gRNAs mapped over than five 1-bp mismatched targets that are likely from multi-targeting effects of 1-bp mismatched targets. C,D) Agreement of genes that have gRNAs targeting 5 of more regions with 1-bp mismatch between Sanger data (Score project data) and Broad data (Avana dataset) A) before multi-targeting effect correction and B) after multi-targeting effect correction.

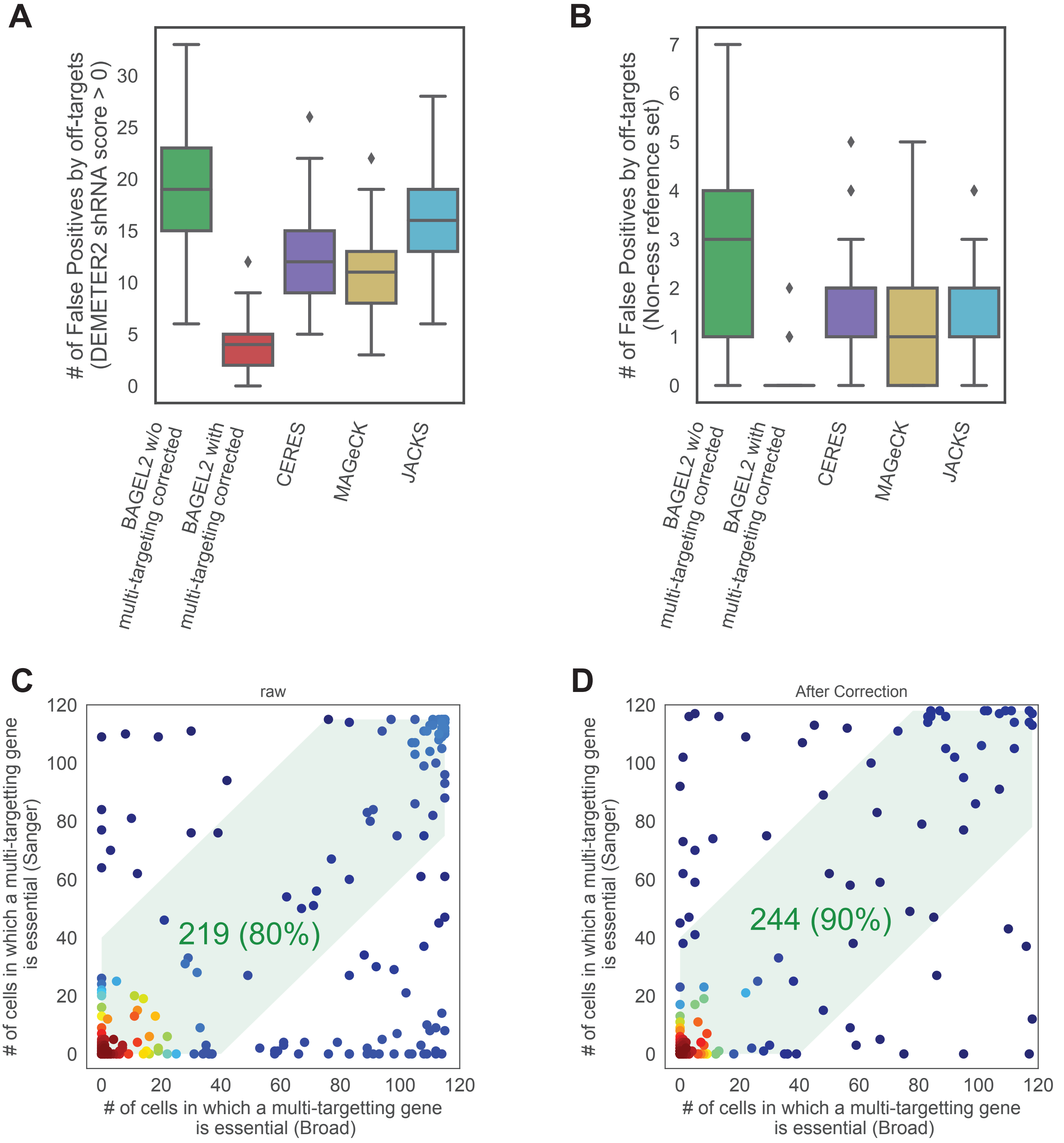

Fig S4. A,B) Fold change distribution plots for a replicate of HUP-T3 cell (A) before and (B) after fold change perturbation. To generate an low performance outlier sample, we added random noise to foldchange value. C) Quality scores of each replicate. D) F-measures (BF = 5) of combination of replicates. Adding the outlier (replicate D) to other high confident replicates reduce overall performance in condition of a few replicates (A vs AD and AB vs ABD).

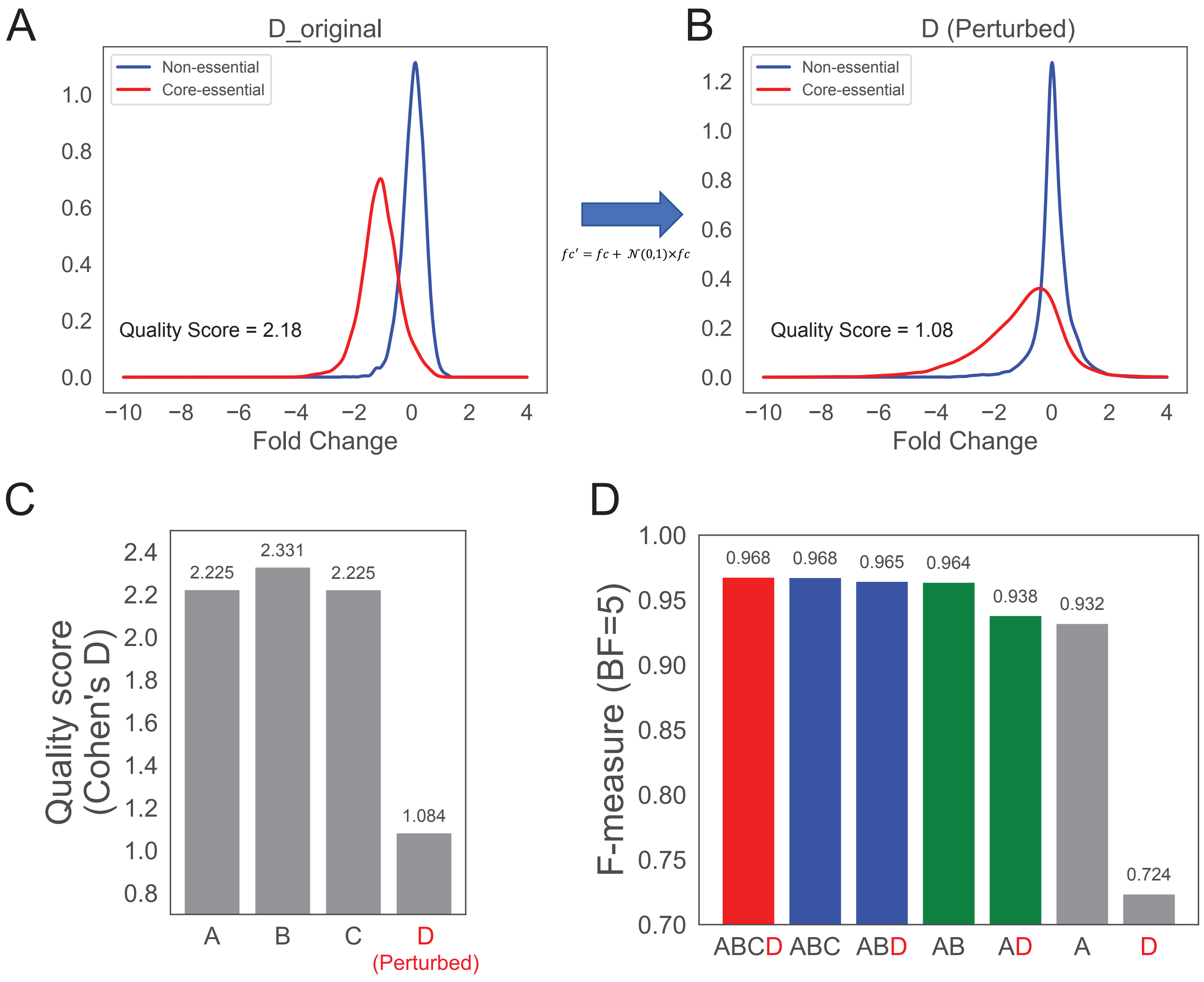

Supplement: Supplementary file 1 — Additional file 1: Fig. S1. A) A brief flow diagram of CRISPR pooled library screen analysis using BAGEL pipeline with additional description about threshold B) A brief scheme of downsampling analysis for defining a log decay threshold function C) A scatter plot represented log-density after down-sampling (50%, 25%, 10%, 5%, 1%) at the original left side x limit, XL0. D) A plot of maximum log density of each down-sampling proportion using DepMap screens and the applied log-decay function in BAGEL2 derived from DepMap Achilles CRISPR screens. Fig. S2. A) A plot explains how to calculate increment of Bayes Factor for measuring multi-targeting effect. B) A two-dimensional dot plot gRNAs targeting multiple regions but only targeting one protein-coding gene. Each dot is located at the number of perfect-matched targets and 1-bp mismatched targets with random jitter and colored by increment of Bayes Factor. Fig. S3. A,B) The number of false positives defined by A) non-essential genes in matched shRNA screens (score > 0, DEMETER2) and B) reference non-essential genes in predicted essential genesets when the scope is limited to genes having gRNAs mapped over than five 1-bp mismatched targets that are likely from multi-targeting effects of 1-bp mismatched targets. C,D) Agreement of genes that have gRNAs targeting 5 or more regions with 1-bp mismatch between Sanger data (Project Score data) and Broad data (Avana dataset) A) before multi-targeting effect correction and B) after multi-targeting effect correction. Fig. S4. A,B) Fold change distribution plots for a replicate of HUP-T3 cell (A) before and (B) after fold change perturbation. To generate an low performance outlier sample, we added random noise to foldchange value. C) Quality scores of each replicate. D) F-measures (BF = 5) of combination of replicates. Adding the outlier (replicate D) to other high confident replicates reduce overall performance in condition of a few replicates (A vs AD and AB vs ABD). [file 13073_2020_809_MOESM1_ESM.pdf]
